# Body mass index trajectories and the risk for Alzheimer’s disease among older adults

**DOI:** 10.1038/s41598-021-82593-7

**Published:** 2021-02-04

**Authors:** Seo Young Kang, Ye-Jee Kim, Wooyoung Jang, Ki Young Son, Hye Soon Park, Young Sik Kim

**Affiliations:** 1 International Healthcare Center, Asan Medical Center, Seoul, Korea; 2 Department of Clinical Epidemiology and Biostatics, Asan Medical Center, Seoul, Korea; 3 Department of Neurology, Gangneung Asan Hospital, University of Ulsan College of Medicine, Seoul, Korea; 4 Department of Family Medicine, Asan Medical Center, University of Ulsan College of Medicine, 88, Olympic-ro-43-gil, Songpa-gu, Seoul, 05505 Korea

**Keywords:** Diseases, Health care, Medical research, Neurology, Risk factors

## Abstract

The effect of body mass index (BMI) changes and variability on the risk for Alzheimer’s disease (AD) remains unclear. We analyzed 45,076 participants, whose BMI were measured on phase 1 (2002–2003), phase 2 (2004–2005), and phase 3 (2006–2007), of the Korean National Health Insurance Service-Health Screening Cohort. We evaluated the effect of 2- and 4-year BMI changes and BMI variability on the risk of AD using Cox regression models. In men, association between 2-year BMI changes, BMI variability, and the risk of AD was not significant. Risk of AD was higher in men whose BMI had decreased 10.1–15.0% over 4 years. In women, aHRs and 95% CIs for AD were 1.14 (1.02–1.29), 1.44 (1.17–1.79), and 1.51 (1.09–2.09) when 2-year BMI loss was 5.1–10.0%, 10.1–15.0%, and > 15.0%. The HRs for AD in women significantly increased when 4-year BMI loss was > 5.0%. The aHR and 95% CI for AD was 1.31 (1.17–1.46) in the 4th quartile of average successive variability (ASV) compared with the 1st quartile of ASV in women. BMI loss over 2- and 4-year period was associated with increased risk for AD, and risk increased in women with higher BMI variability. Appropriate body weight management is recommended to prevent AD.

## Introduction

Alzheimer’s disease (AD), the most common subtype of dementia, is a chronic progressive neurodegenerative disorder characterized by cognitive dysfunction, behavioral disturbances, and difficulties in activities of daily living^1,2^. As the disease progresses, language problems, disorientation, mood swings, and behavioral issues gradually worsen, and ultimately, a patient’s bodily functions are lost^1,2^, leading to a heavy socioeconomic burden in many countries^3,4^. Patients at risk for AD can be identified in primary care settings, as they may complain of mild changes in memory and thinking abilities^2^. Early diagnosis of Alzheimer’s disease leads to appropriate pharmacological intervention and initiation of social support, which could control some of the symptoms^5^. Therefore, early identification of those at risk of developing AD is essential.

Several modifiable risk factors have been proposed for AD although physiological mechanisms, such as cerebral deposition of the amyloid β–protein and hyperphosphorylation of tau protein, are pivotal in disease development^6–8^. Despite controversies, numerous studies have investigated the relationship between body mass index (BMI) and the risk of AD. In general, higher midlife BMI was associated with an increased risk of AD, whereas higher late-life BMI was associated with a reduced risk of AD^9^. The most recent meta-analysis reported that midlife underweight, midlife obesity, and late-life underweight increases overall dementia risk. Particularly, midlife risk for AD increased when BMI > 30 kg/m^2^, and late-life risk for AD decreased when BMI < 27 kg/m^2^^10^.

In addition to examining the effect of single point BMI on the risk of AD, few studies have evaluated the association between BMI trajectories and risk of AD. Studies have evaluated either “change” in BMI, which is a difference between measurements at two time points, or “variability”, which is a fluctuation of BMI among three or more measurements. Generally, BMI or weight loss and higher BMI or weight variability were associated with an increased risk for AD^11–16^. These studies concluded that weight loss precedes AD since weight loss may be a causal factor or a prodromal symptom of AD^11–14^. Furthermore, recent evidence suggests that a loss of physiological homeostasis can lead to variability in metabolic parameters, ultimately causing disease^17,18^.

To our knowledge, there is only one study that evaluated the association between BMI change and risk of dementia in the general Korean population. Unlike previous studies, this study reported that both BMI gain and loss were associated with an increased risk for overall dementia^19^. However, the outcome variable in this study was not restricted to AD, and the definition of dementia solely relied on the International Classification of Diseases, 10th edition (ICD-10), which is subject to false positive. Therefore, in this study, we investigated the association between BMI changes and risk for AD after defining AD based on a combination of the ICD-10 codes and antidementia drug prescription in a nationwide representative sample. Based on three BMI measurements over 4 years, we evaluated 2- and 4-year BMI changes and BMI variability to investigate the effect of BM trajectories on the risk of AD.

## Methods

### Data source and study population

We used data from the National Health Insurance Service-Health Screening Cohort (NHIS-HEALS). In Korea, NHIS provides compulsory health insurance covering approximately 97% of the entire population^20^. Furthermore, NHIS provides biennial health screening to all insured individuals. NHIS-HSALS is an anonymized academic database, consisting of a random selection of 10% of all examinees who participated in the biennial national health screening program during 2002 and 2003 (baseline year/phase 1)^21^. It includes 514,866 participants, aged between 40 and 79 at the baseline year, who were followed up to the year 2015. NHIS-HEALS comprises 4 databases on the participants' insurance eligibility, medical counseling, health screening, and medical care institutions. Insurance eligibility database contains details on demographics, death-related information, type of health insurance, and household income. Medical counseling database offers details on diagnosed diseases based on ICD-10 and prescription-related information. Health screening database provides results of biennial health screening, such as assessment of health behaviors, anthropometric measurements, and laboratory results of blood and urine samples. Medical care institutions database provides information for each medical care facility.

From the 514,866 participants, we excluded participants aged < 60 years at the baseline year (N = 382,013) (Figure S1). Among 132,853 participants aged ≥ 60 years, we excluded 6045 participants diagnosed with any type of dementia; 15,608 participants died before the index date (January 1st, 2011). We further excluded 1811 cancer patients and participants whose BMI was not measured in any of the three consecutive health screenings and whose lifestyle variables were not measurable due to missing responses, leaving 45,076 participants for the final analysis.

### Ethics declarations

The study protocol was approved by the Institutional Review Board of Asan Medical Center (IRB number 2018-1524). All research procedures were employed in accordance with the relevant guidelines and regulations. As the NHIS-HEALS database is comprised of de-identified secondary data, obtaining written informed consent from study participants was unnecessary.

### Definition of AD

We assessed AD based on the codes from ICD-10. Participants who were prescribed choline acetyltransferase inhibitor (donepezil, galantamine, rivastigmine) or NMDA receptor antagonist (memantine) at least twice with ICD-10 codes pertaining to AD (F00, G30) were determined to have developed AD. In order to fulfill Korean National Health Insurance reimbursement criteria, physicians need to document the evidence for cognitive dysfunction according to the following criteria: (1) Mini Mental State Examination (MMSE) score ≤ 26 and (2) either a Clinical Dementia Rating (CDR) score ≥ 1 or a Global Deterioration Scale (GDS) score ≥ 3^22^. Dementia was diagnosed when participants were prescribed the above antidementia drug at least twice with ICD-10 codes pertaining to dementia (F00, F01, F02, F03, G30, G31), and subjects with dementia were excluded when diagnosed prior to the index date.

### Changes in BMI

BMI was calculated by dividing body weight in kilograms by the square of height in meters. BMI was further categorized into four groups: < 18.5 kg/m^2^, 18.5–22.9 kg/m^2^, 23.0–24.9 kg/m^2^, ≥ 25.0 kg/m^2^. BMI changes over 2 years was defined as percent difference between baseline BMI (phase 1: 2002–2003) and that measured after 2 years (phase 2: 2004–2005). BMI changes over 4 years was defined as the percent difference between BMI measured at baseline and after 4 years (phase 3: 2006–2007). Percent BMI changes were further categorized into the following 7 groups: stable ± 5.0%, decrease of 5.1–10.0%, decrease of 10.1–15.0%, decrease of > 15.0%, increase of 5.1–10.0%, increase of 10.1–15.0%, and increase of > 15.0%.

### BMI variability

BMI variability was determined using average successive variability (ASV), which was defined as the average absolute difference between BMI values of the three phases of health screening^23^. ASV was further divided into quartiles.

### Other variables

Covariates were assessed from the baseline year. Sociodemographic variables included age and insurance premium. Lifestyle factors were evaluated, including smoking status, alcohol consumption, and regular exercise. Smoking status was categorized as non-smokers and smokers. Alcohol consumption was categorized as non-, moderate, and heavy drinker, according to the definitions of the National Institute on Alcohol Abuse and Alcoholism^24^. Regarding regular exercise, participants were asked to provide weekly frequency of exercise. We categorized participants as those who were engaging in exercise ≥ 1 per week (yes) and those who were not (no). We assessed presence of hypertension, diabetes mellitus, and dyslipidemia based on codes from ICD-10. If insurance claims for either outpatient or inpatient treatment were made at least 3 times with ICD-10 codes pertaining to a certain comorbidity, we determined that participant had the comorbidity. The ICD-10 codes were as follows: hypertension (I10, I11, I12, I13, I15), diabetes mellitus (E10, E11, E12, E13, E14), and dyslipidemia (E78).

### Statistical analysis

Participants were followed up from the index date to the date AD was diagnosed, date of death, or December 31st, 2015, whichever came first. To reduce the possibility of reverse causation, the index date was set to January 1st, 2011, with 3-year latent period for the diagnosis of AD from the last BMI measurement. We used chi-square test to compare baseline characteristics between men and women. To evaluate the effect of 2- and 4-year BMI changes on the risk of AD, we performed Cox proportional hazards regression analysis after adjusting for age, insurance premium, initial BMI, smoking status, alcohol consumption, physical activity, hypertension, diabetes mellitus, and dyslipidemia. Hazard ratios (HRs) and 95% confidence intervals (CIs) for AD were obtained for each category of BMI changes, considering “stable (± 5.0%)” as a reference group. We plotted a restricted cubic spline of HRs for AD on a logarithmic scale to examine the association using 2- and 4-year BMI changes as continuous variables. To evaluate the effect of BMI variability on the risk of AD, we performed Cox proportional hazards regression analysis, where ASV was computed as both a continuous variable and a categorical variable. We obtained the HRs and 95% CIs for AD for 1 ASV change and in each ASV quartile, considering the 1st quartile as a reference group. Subgroup analyses were performed based on lifestyle factors in order to evaluate the risk for AD according to BMI changes and variability in each category of lifestyle factors. Analyses were separately performed for men and women. Analyses were conducted using SAS version 9.4 (SAS Institute, Cary, NC, USA), and restricted cubic splines were plotted using rms package in R software version 3.3.3 (R Foundation for Statistical Computing, Vienna, Austria). Two-tailed p values < 0.05 were considered statistically significant.

## Results

### Baseline characteristics, BMI changes, and BMI variability of the study participants

Among the 45,076 study participants (23,298 men and 21,778 women), 4055 cases (1688 men and 2367 women) of AD were observed during the 2.7-year mean follow-up. The cumulative incidence rate for AD was 16.3 cases per 1000 person-years in men and 23.9 cases per 1000 person-years in women. Table 1 shows baseline characteristics, BMI changes, and BMI variability of study participants. Men and women were equally distributed among age groups. The proportion of obese individuals was higher in women, while the proportion of current smokers and heavy drinkers was higher in men. The prevalence of lack of regular exercise, hypertension, diabetes mellitus, and dyslipidemia was higher in women. Approximately 73.3% of men and 68.6% of women maintained stable BMI over 2 years, and 66.1% of men and 62.6% of women maintained stable BMI over 4 years. The proportions of participants were lower in categories with greater BMI changes. ASVs of the BMI for the 3 phases of health screening were 1.44 ± 1.23 kg/m^2^ in men and 1.63 ± 1.32 kg/m^2^ in women.

**Table 1:** Baseline characteristics, BMI changes, and BMI variability of study participants (n = 45,076).

	Men (N = 23,298)	Women (N = 21,778)	p-value
	N (%)	N (%)	
**Age (years)**			
60–69	19,222 (82.5)	17,960 (82.5)	0.921
70–79	4,076 (17.5)	3,818 (17.5)	
**Insurance premium (deciles**)			
≤ 2 (low)	4437 (19.0)	4180 (19.2)	0.008
3–5	5693 (24.4)	5069 (23.3)	
6–8	6543 (28.1)	6080 (27.9)	
9–10 (high)	6625 (28.4)	6449 (29.6)	
**BMI (kg/m**^**2**^**)**			
< 18.5	776 (3.3)	527 (2.4)	< 0.001
18.5–22.9	9063 (38.9)	6845 (31.4)	
23–24.9	6512 (28.0)	5560 (25.5)	
≥ 25	6947 (29.8)	8846 (40.6)	
**Smoking status**			
Non-smoker	16,351 (70.2)	21,194 (97.3)	< 0.001
Current smoker	6947 (29.8)	584 (2.7)	
**Alcohol consumption**			
Non-drinker	10,505 (45.1)	19,644 (90.2)	< 0.001
Moderate drinker	9555 (41.0)	1841 (8.5)	
Heavy drinker	3238 (13.9)	293 (1.3)	
**Regular exercise**			
No	12,526 (53.8)	15,724 (72.2)	< 0.001
Yes	10,772 (46.2)	6054 (27.8)	
**Comorbidities**			
Hypertension	6810 (29.2)	7475 (34.3)	< 0.001
Diabetes mellitus	2553 (11.0)	2587 (11.9)	< 0.001
Dyslipidemia	1675 (7.2)	2300 (10.6)	< 0.001
**2-year BMI changes**			
Decrease of > 15.0%	156 (0.7)	238 (1.1)	< 0.001
Decrease of 10.1–15.0%	441 (1.9)	595 (2.7)	
Decrease of 5.1–10.0%	2676 (11.5)	2808 (12.9)	
Stable ± 5.0%	17,085 (73.3)	14,941 (68.6)	
Increase of 5.1–10.0%	2251 (9.7)	2344 (10.8)	
Increase of 10.1–15.0%	455 (2.0)	558 (2.6)	
Increase of > 15.0%	234 (1.0)	294 (1.3)	
**4-year BMI changes**			
Decrease of > 15.0%	240 (1.0)	328 (1.5)	< 0.001
Decrease of 10.1–15.0%	659 (2.8)	779 (3.6)	
Decrease of 5.1–10.0%	3,075 (13.2)	3,057 (14.0)	
Stable ± 5.0%	15,394 (66.1)	13,632 (62.6)	
Increase of 5.1–10.0%	2871 (12.3)	2780 (12.8)	
Increase of 10.1–15.0%	736 (3.2)	817 (3.8)	
Increase of > 15.0%	323 (1.4)	385 (1.8)	
**BMI variability**			
Average successive variability^a^ (kg/m^2^)	1.44 ± 1.23	1.63 ± 1.32	< 0.001

*BMI* body mass index.

^a^Values are presented as mean ± standard deviation.

### BMI changes and risk for AD

**Figure 1:**
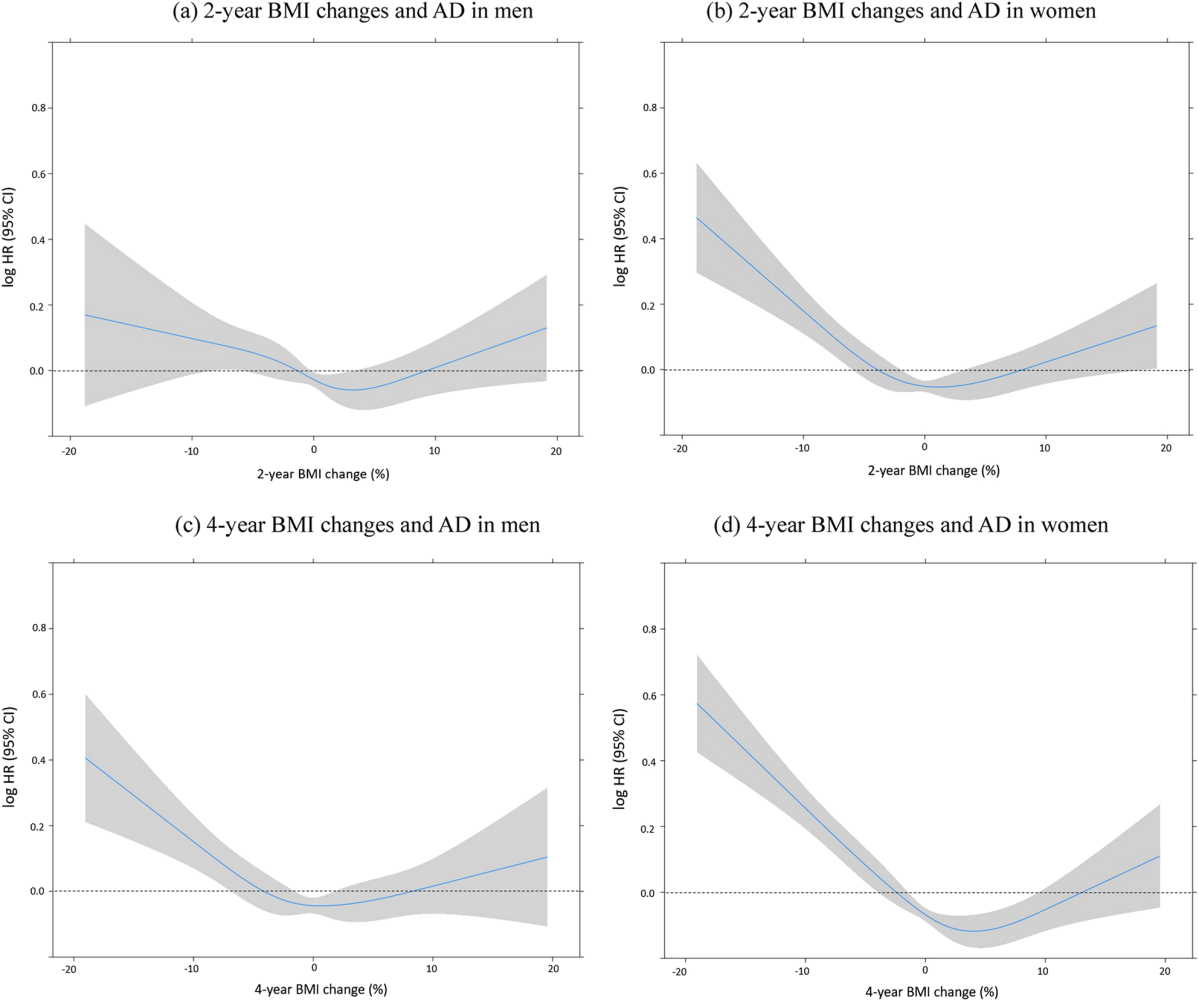
BMI changes and risk of Alzheimer’s disease.

Table 2 shows HRs and 95% CIs for AD in each category of BMI changes in the 2- and 4-year periods in comparison to the stable BMI group. In the multivariate model of men, association between 2-year BMI changes and risk of AD was not significant. Risk of AD was 1.33 (95% CI 1.02–1.72) higher in men whose BMI had decreased 10.1–15.0% over 4 years. In spline representation of logarithmic HR for AD, the association between 2-year BMI changes and risk for AD was not evident (Fig. 1). With the lowest risk in 0–1% gain in BMI, logarithmic HR for AD increased as 4-year BMI changes decreased. Risk of AD was associated with BMI loss in women. Adjusted hazard ratios (aHRs) and 95% CIs for AD were 1.14 (1.02–1.29), 1.44 (1.17–1.79), and 1.51 (1.09–2.09) in women whose 2-year BMI loss was 5.1–10.0%, 10.1–15.0%, and > 15.0%, respectively. The aHRs and 95% CIs for AD were 1.31 (1.17–1.46), 1.60 (1.33–1.92), and 1.68 (1.29–2.20) in women whose 4-year BMI loss was 5.1–10.0%, 10.1–15.0%, and > 15.0%, respectively. In spline representation of logarithmic HR for AD, the association between 2- and 4-year BMI changes and the risk for AD showed a U-shape with the lowest risk in 0–2% and 3–5% BMI gain, respectively (Fig. 1). The increase in the risk for AD was only significant when BMI decreased.

**Table 2:** Risk of Alzheimer’s disease according to BMI changes.

	Men					Women				
	Event	Duration		Unadjusted HR (95% CI)	^a^Adjusted HR (95% CI)	Event	Duration		Unadjusted HR (95% CI)	^a^Adjusted HR (95% CI)
	N	Person-years	CIR			N	Person-years	CIR		
**2-year BMI changes**										
Decrease of > 15.0%	11	670	16.4	1.04 (0.58–1.89)	1.00 (0.55–1.82)	38	1034	36.8	**1.64 (1.19–2.26)**	**1.51 (1.09–2.09)**
Decrease of 10.1–15.0%	39	1865	20.9	1.34 (0.97–1.84)	1.21 (0.88–1.66)	91	2614	34.8	**1.54 (1.25–1.91)**	**1.44 (1.17–1.79)**
Decrease of 5.1–10.0%	220	11,682	18.8	**1.20 (1.04–1.39)**	1.14 (0.98–1.31)	335	12,653	26.5	**1.17 (1.04–1.32)**	**1.14 (1.02–1.29)**
Stable (± 5.0%)	1203	76,364	15.8	1.00	1.00	1,543	68,107	22.7	1.00	1.00
Increase of 5.1–10.0%	162	9869	16.4	1.05 (0.89–1.23)	0.95 (0.80–1.12)	240	10,728	22.4	0.99 (0.86–1.13)	0.94 (0.82–1.08)
Increase of 10.1–15.0%	35	1955	17.9	1.14 (0.82–1.60)	1.00 (0.71–1.40)	77	2485	31.0	**1.37 (1.09–1.72)**	1.17 (0.93–1.47)
Increase of > 15.0%	18	1034	17.4	1.11 (0.70–1.76)	0.88 (0.55–1.41)	43	1285	33.5	**1.49 (1.10–2.02)**	1.17 (0.86–1.59)
**4-year BMI changes**										
Decrease of > 15.0%	19	1020	18.6	1.21 (0.77–1.90)	1.14 (0.73–1.80)	56	1360	41.2	**1.92 (1.47–2.51)**	**1.68 (1.29–2.20)**
Decrease of 10.1–15.0%	60	2782	21.6	**1.39 (1.08–1.81)**	**1.33 (1.02–1.72)**	126	3409	37.0	**1.72 (1.43–2.06)**	**1.60 (1.33–1.92)**
Decrease of 5.1–10.0%	245	13,426	18.2	**1.18 (1.02–1.35)**	1.13 (0.98–1.30)	405	13,592	29.8	**1.38 (1.23–1.54)**	**1.31 (1.17–1.46)**
Stable (± 5.0%)	1,071	68,805	15.6	1.00	1.00	1,354	62,410	21.7	1.00	1.00
Increase of 5.1–10.0%	200	12,803	15.6	1.00 (0.86–1.17)	0.96 (0.83–1.12)	284	12,723	22.3	1.03 (0.91–1.17)	0.98 (0.87–1.12)
Increase of 10.1–15.0%	69	3205	21.5	**1.39 (1.09–1.77)**	1.21 (0.94–1.55)	91	3716	24.5	1.13 (0.91–1.40)	1.00 (0.80–1.23)
Increase of > 15.0%	24	1398	17.2	1.11 (0.74–1.66)	0.88 (0.58–1.32)	51	1697	30.1	**1.40 (1.06–1.85)**	1.13 (0.85–1.50)

*BMI* body mass index, *CIR* cumulative incidence rate, *HR* hazard ratio, *CI* confidence interval.

^a^Adjusted for age, insurance premium, initial BMI, smoking status, alcohol consumption, regular exercise, hypertension, diabetes mellitus, and dyslipidemia.

### BMI variability and risk for AD

Table 3 shows risk of AD based on BMI variability in the three phases of health screening. In the multivariate model of men, association between BMI variability and risk of AD was not significant. In women, the risk of AD increased approximately 6% when ASV changed 1 kg/m^2^. The aHRs and 95% CIs for AD was 1.31 (1.17–1.46) in the 4th quartile of ASV compared with the 1st quartile.

**Table 3:** Risk of Alzheimer’s disease according to BMI variability.

	Men					Women				
	Event	Duration		Unadjusted HR (95% CI)	^a^Adjusted HR (95% CI)	Event	Duration		Unadjusted HR (95% CI)	^a^Adjusted HR (95% CI)
	N	Person-years	CIR			N	Person-years	CIR		
**BMI variability**										
ASV	–	**–**	**–**	**1.05 (1.03–1.08)**	1.05 (1.02–1.08)	–	**–**	**–**	**1.07 (1.05–1.09)**	**1.06 (1.03–1.08)**
BMI variability										
ASV 1st quartile	404	26,149	15.4	1.00	1.00	520	24,913	20.9	1.00	1.00
ASV 2nd quartile	383	25,998	14.7	0.95 (0.83–1.10)	0.93 (0.81–1.07)	532	25,027	21.3	1.02 (0.90–1.15)	1.01 (0.89–1.13)
ASV 3rd quartile	412	25,846	15.9	1.03 (0.90–1.19)	0.98 (0.86–1.13)	595	**24,626**	**24.2**	**1.16 (1.03–1.30)**	1.10 (0.98–1.24)
ASV 4th quartile	489	25,446	19.2	**1.25 (1.09–1.42)**	1.13 (0.99–1.29)	720	**24,341**	**29.6**	**1.42 (1.27–1.59)**	**1.31 (1.17–1.46)**

*BMI* body mass index, *ASV* average successive variability, *CIR* cumulative incidence rate, *HR* hazard ratio, *CI* confidence interval.

^a^Adjusted for age, insurance premium, initial BMI, smoking status, alcohol consumption, regular exercise, hypertension, diabetes mellitus, and dyslipidemia.

### Subgroup analysis

Table 4 shows the risk for AD among participants with 2-year BMI loss > 5.0%, 4-year BMI loss > 5.0%, and 4th quartile of ASV in each category of lifestyle factors. In multivariate model of men, 2-year BMI loss did not generally increase the risk for AD; however, the risk for AD increased among current smokers and heavy drinkers when their BMI had decreased > 5.0% over the past 2 years. The risk for AD was 1.16 (95% CI 1.03–1.32) higher when 4-year BMI loss was > 5.0%, and this association was significant among obese men and those who were not doing regular exercise. More than 5.0% of BMI gain over the 2- and 4-year periods did not increase risk for AD in men (data not shown). The risk for AD in the 4th ASV quartile compared with the 1^st^ was higher among obese men and moderate drinkers. In multivariate model of women, the risk for AD increased in those with 2-year BMI loss > 5.0%, 4-year BMI loss > 5.0%, and those whose ASV of BMI was in the highest quartile. The association was significant among normal, overweight, and obese women, as well as non-smokers, non-drinkers, and regardless of regular exercise. More than 5.0% of BMI gain over 2-year and 4-year period did not increase the risk for AD in women (data not shown).

**Table 4:** Subgroup analysis for association between 2-year BMI loss, 4-year BMI loss, BMI variability and risk for Alzheimer’s disease according to lifestyle factors.

	Men		Women	
	Unadjusted HR (95% CI)	^a^Adjusted HR (95% CI)	Unadjusted HR (95% CI)	^a^Adjusted HR (95% CI)
**2-year BMI loss > 5.0% vs. stable (± 5.0%) BMI**				
All	**1.21 (1.06–1.38)**	1.14 (0.99–1.30)	**1.26 (1.14–1.40)**	**1.22 (1.10–1.35)**
Underweight	0.67 (0.27–1.69)	0.49 (0.19–1.24)	1.25 (0.59–2.68)	1.26 (0.58–2.70)
Normal and overweight	**1.26 (1.07–1.49)**	1.18 (0.99–1.39)	**1.28 (1.11–1.47)**	**1.22 (1.05–1.40)**
Obesity	1.19 (0.95–1.50)	1.12 (0.89–1.40)	1.26 (1.08–1.48)	**1.22 (1.04–1.42)**
Non-smoker	1.16 (0.99–1.35)	1.08 (0.93–1.27)	**1.26 (1.13–1.40)**	**1.22 (1.10–1.36)**
Current smoker	**1.35 (1.06–1.72)**	**1.29 (1.01–1.64)**	1.22 (0.71–2.11)	1.06 (0.61–1.87)
Non-drinker	1.08 (0.89–1.31)	1.05 (0.87–1.28)	**1.25 (1.12–1.39)**	**1.21 (1.08–1.35)**
Moderate drinker	1.21 (0.96–1.52)	1.11 (0.88–1.40)	1.41 (0.99–2.01)	1.36 (0.95–1.94)
Heavy drinker	**1.50 (1.11–2.01)**	**1.43 (1.07–1.93)**	1.19 (0.50–2.85)	1.12 (0.46–2.76)
No regular exercise	**1.22 (1.03–1.44)**	1.18 (0.99–1.39)	**1.25 (1.11–1.40)**	**1.21 (1.08–1.36)**
Regular exercise	1.17 (0.94–1.44)	1.08 (0.87–1.33)	1.24 (0.99–1.55)	**1.26 (1.00–1.57)**
**4-year BMI loss > 5.0% vs. stable (± 5.0%) BMI**				
All	**1.21 (1.07–1.37)**	**1.16 (1.03–1.32)**	**1.48 (1.34–1.63)**	**1.39 (1.26–1.53)**
Underweight	0.59 (0.18–1.91)	0.50 (0.15–1.63)	**2.23 (1.15–4.30)**	1.86 (0.95–3.65)
Normal and overweight	1.15 (0.98–1.35)	1.08 (0.92–1.27)	**1.38 (1.21–1.58)**	**1.28 (1.12–1.47)**
Obesity	**1.41 (1.15–1.73)**	**1.36 (1.11–1.66)**	**1.62 (1.41–1.88)**	**1.52 (1.31–1.75)**
Non-smoker	**1.22 (1.06–1.41)**	1.15 (0.99–1.33)	**1.48 (1.34–1.64)**	**1.41 (1.27–1.55)**
Current smoker	1.19 (0.94–1.51)	1.18 (0.93–1.50)	1.36 (0.79–2.34)	0.98 (0.55–1.72)
Non-drinker	1.14 (0.96–1.37)	1.11 (0.92–1.32)	**1.50 (1.35–1.66)**	**1.41 (1.27–1.56)**
Moderate drinker	**1.25 (1.01–1.54)**	1.22 (0.98–1.50)	**1.53 (1.09–2.16)**	1.41 (0.99–1.99)
Heavy drinker	1.33 (0.98–1.80)	1.22 (0.90–1.66)	0.55 (0.22–1.37)	0.44 (0.16–1.20)
No regular exercise	**1.30 (1.11–1.53)**	**1.26 (1.07–1.48)**	**1.41 (1.27–1.58)**	**1.34 (1.20–1.50)**
Regular exercise	1.10 (0.90–1.33)	1.04 (0.86–1.27)	**1.66 (1.35–2.04)**	**1.59 (1.30–1.96)**
**ASV 4th quartile vs. 1st quartile**				
All	**1.25 (1.09–1.42)**	1.13 (0.99–1.29)	**1.42 (1.27–1.59)**	**1.31 (1.17–1.46)**
Underweight	0.89 (0.47–1.66)	0.79 (0.42–1.51)	1.17 (0.62–2.22)	0.99 (0.51–1.90)
Normal and overweight	**1.19 (1.01–1.40)**	1.06 (0.91–1.25)	**1.44 (1.24–1.67)**	**1.31 (1.13–1.52)**
Obesity	**1.50 (1.16–1.92)**	**1.38 (1.07–1.78)**	**1.45 (1.21–1.74)**	**1.33 (1.11–1.59)**
Non-smoker	**1.28 (1.09–1.50)**	1.16 (0.99–1.36)	**1.42 (1.27–1.60)**	**1.32 (1.17–1.48)**
Current smoker	1.17 (0.92–1.49)	1.07 (0.84–1.36)	1.30 (0.68–2.48)	1.00 (0.52–1.95)
Non-drinker	1.17 (0.97–1.41)	1.10 (0.91–1.33)	**1.41 (1.26–1.59)**	**1.30 (1.15–1.47)**
Moderate drinker	**1.40 (1.12–1.75)**	**1.26 (1.01–1.58)**	1.45 (0.99–2.12)	1.28 (0.87–1.88)
Heavy drinker	1.06 (0.76–1.48)	0.97 (0.70–1.36)	2.32 (0.66–8.24)	2.30 (0.62–8.56)
No regular exercise	**1.19 (1.00–1.41)**	1.11 (0.93–1.31)	**1.32 (1.16–1.50)**	**1.25 (1.10–1.43)**
Regular exercise	**1.25 (1.02–1.55)**	1.16 (0.94–1.43)	**1.70 (1.34–2.16)**	**1.50 (1.18–1.92)**

*BMI* body mass index, *HR* hazard ratio, *CI* confidence interval, *ASV* average successive variability.

^a^Adjusted for age, insurance premium, initial BMI, smoking status, alcohol consumption, regular exercise, hypertension, diabetes mellitus, and dyslipidemia.

## Discussion

BMI loss over the 2- and 4-year periods increased the risk for AD. Specifically, the risk increased in women as the amount of BMI loss over both time periods had increased. In men, this association was not clear on multivariate analysis; however, the risk tended to increase when amount of BMI loss over the 4-year period had increased. In contrast, BMI gain was not associated with increased risk for AD. Additionally, higher BMI variability increased the risk for AD in women.

The association between weight loss and the risk of AD can be explained by the following physiological mechanisms. As weight loss is a byproduct of low energy intake, subsequent deficiency in macronutrients and micronutrients may aggravate cognitive function^25,26^. For instance, deficiency in essential fatty acids comprising neuronal cell membrane results in physiological changes in the neuronal cell membrane, and vitamin deficiency may aggravate oxidative stress^26,27^. Furthermore, weight loss contributes to cognitive impairment by raising the levels of serum cortisol and free radicals^25,28^. Moreover, weight loss decreases the amount of leptin produced from subcutaneous and visceral fat tissue^29^. Leptin is a hormone, which stimulates axonal growth, synaptogenesis, and cell survival, protects against oxidative stress, and associated with hippocampal progenitor cell proliferation^29^. Thus, decrease in leptin effect could lead to cognitive decline. In the Framingham study, people with the lowest quartile of leptin levels had a four-time greater risk of developing AD after 12 years than those with the highest quartile of leptin level^30^.

As our analysis is focused on adults ≥ 60 years, bone loss and sarcopenia are critical factors linked to weight loss. Decline in bone mass may increase inflammatory markers, which also increase the risk for AD^31^. In addition, predisposing conditions for sarcopenia, such as insulin resistance, inflammation, and oxidative stress, are also associated with cognitive dysfunction^32^. Although some of these mechanisms remain uncertain, studies have shown the link between bone loss, sarcopenia, and cognitive impairment^33–35^. Therefore, regular physical activity with strength exercise is recommended to prevent cognitive impairment in the elderly population.

Despite the forementioned biologic mechanism suggesting weight loss can be causative for development of AD, weight loss can also be prodromal or early symptoms of AD. Atrophy of the brain areas that play a role in weight control, such as the mesial temporal cortex, may occur during the preclinical phase of AD, thereby leading to weight loss^36^. Similarly, hypometabolism of the hypothalamus or cingulate gyrus may impair weight control and contribute to weight loss prior to the development of AD^37^. Furthermore, early symptoms of AD such as reduced olfactory function and predementia apathy may cause weight loss^2^. Therefore, studies have supported the argument for weight loss presenting as a prodromal symptom of AD rather than a risk factor. However, recent studies have shown that weight loss may even precede mild cognitive impairment, and weight loss was associated with biomarkers for AD in healthy individuals, as well as baseline cortical thinning and accelerated brain atrophy^38,39^. In our study, BMI was measured three times, in 2002–2003, 2004–2005, and 2006–2007, and the development of AD was measured from January 1, 2011, with a minimum 5 year gap for the association between 2-year BMI change and risk for AD, and at least a 3 year gap for the association between 4-year BMI change and risk for AD. Given the chronic and neurodegenerative nature of AD, the latent period in our study may not be sufficient to exclude the possibility of reverse causation. Nevertheless, it is very clear from our results that BMI loss precedes the diagnosis of AD by several years.

In our analysis, BMI loss over the 2- and 4-year periods increased the risk for AD in women. This association was not significant in men, although an increasing trend for risk of AD was observed from the cubic spline when a 4-year BMI loss was present. Prevalence of AD was higher among women, and clinical progression and neurodegeneration more rapidly manifested in women than in men^40^. One of possible explanation can be interaction between apolipoprotein E (APOE) ε4 allele polymorphism and sex. APOE ε4 allele, a risk factor for AD, is associated with a greater increase in the probability of developing AD in women than in men^41^. Cognitive and functional decline were faster in women than in men, and this was greater in APOE ε4 allele carriers^42^. Furthermore, women experience a sudden decline in estrogen due to menopause in their early 50 s. Estrogen protects against mitochondrial dysfunction, modulates neurogenesis, and decreases the level of amyloid β-protein^43–45^. Thus, a rapid decrease in estrogen may increase susceptibility for AD in perimenopausal women. Along with genetic and environmental interactions, several risk factors have greater impact on women in the development and progression of AD^40,46^. Likewise, the sex difference observed in this study could be due to the difference in AD susceptibility.

In the subgroup analyses of women, a BMI loss > 5.0% over a 2-year and 4-year period consistently increased risk for AD in general, except for underweight women, current smokers, moderate drinkers, and heavy drinkers. It is more likely due to the low statistical power in these groups of participants, as the proportion of these participants among the entire group of women participants was 2.4%, 2.7%, 8.5%, and 1.3%, respectively. In the main analyses, association between BMI loss and AD among men was only significant when their BMI had decreased 10.1–15.0% over 4 years. However, when participants with a 4-year BMI loss of > 5.0% were placed into one category, the risk for AD increased by 1.16 (95% CI 1.03–1.22). This result, along with the cubic spline showing the association between 4-year BMI change and logarithmic HR, presents an increased risk for AD among men with BMI loss compared with those with stable BMI. However, inconsistent findings from the subgroup analyses do not support this association, and it is difficult to draw a definite conclusion from our analysis for men.

In our study, higher BMI variability increased the risk for AD in women. Recent studies have demonstrated the association between BMI variability and an increased risk for cardiovascular morbidity and mortality^17^. Although the underlying mechanism remains unclear, fluctuation in body weight contributes to fluctuations in blood pressure, heart rate, glomerular filtration rate, and glucose and lipid levels, thereby increasing the risk for cardiovascular morbidity and mortality^47^. Our findings suggest a potential link between weight fluctuation and an increased risk for cognitive decline in addition to cardiovascular risk factors. To our knowledge, there are very few studies, which investigated the association between body weight variability and risk for dementia. Previous studies reported that higher weight variability was associated with an increased risk for dementia^15,16^. In our study, we used ASV, the average absolute difference between BMI values of each measurement, as a measure of variability. In the first part of our analysis, we reported association between BMI loss and increased risk for AD. An increased risk for AD in those with higher ASV of BMI in the same study population additionally shows that BMI instability may also increase risk for AD regardless of the direction of BMI change.

There are several limitations in this study. First, the diagnosis of AD was not made clinically, using ICD-10 codes instead. To overcome this limitation, we defined AD based on the combination of the ICD-10 codes and antidementia drug prescription as patients must have MMSE score ≤ 26, and either a CDR score ≥ 1 or a GDS score ≥ 3 to necessitate such a prescriptions^22^, a large number of participants were excluded due to missing data, which could result in a selection bias. The low participation rate for national health screening in the early 2000s (less than 50% of the Korean population) may have caused the large number of missing values in the three phases of health screening. Third, the mechanisms linking BMI declines and AD remain unexplained as we could not obtain bone density, muscle mass, or abdominal circumference from the dataset. The effect of changes in bone density, muscle mass, and adiposity on the risk for AD will provide better insights for the underlying mechanisms. Fourth, we cannot distinguish intentional from unintentional weight loss among study participants. As our study participants were aged ≥ 60, it is likely that a high proportion of observed weight loss was unintentional while some intentional weight loss may present within this cohort. Furthermore, there were no measures of calorie consumption; thus, it is not possible to determine if the weight loss was due to reduced calorie intake or an endogenous metabolic abnormality. In fact, weight loss appears in pre-clinical stages of many neurodegenerative diseases, suggesting the possibility of behavior shifts and/or energy imbalance in neurodegeneration^25,48,49^. Fifth, we were not able to consider genetic factors or brain functions in this study. Especially, we could not adjust APOE ε4 allele although it is an established genetic risk factor for AD^1,2^. Future research should focus on the potential role of hypothalamic dysfunction as this brain region is an early target in AD^1,2^. Lastly, as AD is a slowly progressing neurodegenerative disease, there is a possibility of reverse causation although we established a latent period of at least 3 years from the last BMI measurement to development of AD. Nevertheless, this is the first study to investigate the effect of BMI changes over different time periods, as well as BMI variability, on risk for AD in the same population. It is also meaningful to report the difference in the effect of BMI trajectories and risk for AD in men and women.

In conclusion, BMI loss over 2- and 4-year periods increased the risk for AD, which also increased in those with higher BMI variability. These associations were significant in women. Appropriate body weight management as well as regular monitoring of body weight and BMI is recommended to prevent AD in the elderly population.

## Supplementary Information

Supplementary Figure S1.

## Data Availability

The data that support the findings of this study are available from National Health Insurance Service-Health Screening Cohort (NHIS-HEALS) database. To gain access, interested individuals should contact Korea National Health Insurance Service.
