# Supplementary material for: Body mass index trajectories and the risk for Alzheimer’s disease among older adults
Source: Sci Rep. 2021 Feb 4;11:3087. doi: 10.1038/s41598-021-82593-7 (PMC7862316; doi:10.1038/s41598-021-82593-7)

**Supplementary Information**

**Title: Body mass index trajectories and the risk for Alzheimer’s disease among older adults**

**Seo Young Kang^1^, Ye-Jee Kim^2^, Wooyoung Jang^3^, Kiyoung Son^4^, Hye Soon Park^4^, Young Sik Kim^4*^**

^1^International Healthcare Center, Asan Medical Center

^2^Department of Clinical Epidemiology and Biostatics, Asan Medical Center

^3^Department of Neurology, Gangneung Asan Hospital, University of Ulsan College of Medicine

^4^Department of Family Medicine, Asan Medical Center, University of Ulsan College of Medicine

Figure S1. Flow of the study population


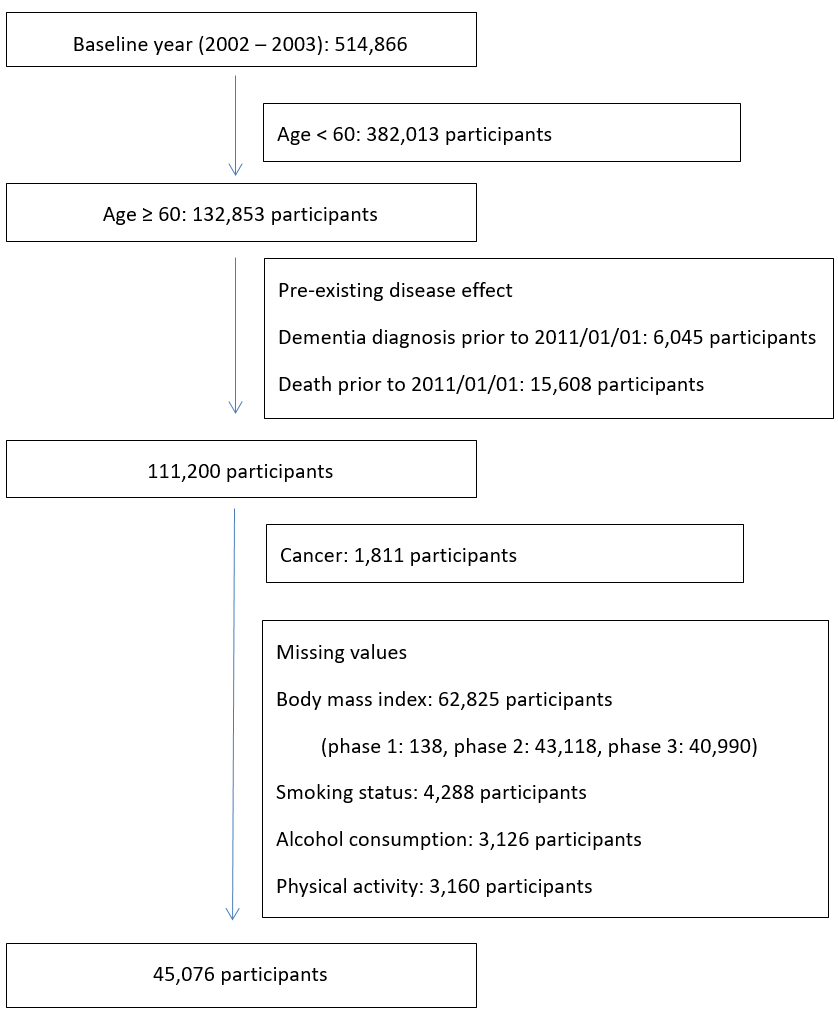

Supplement: Supplementary file 1 — Supplementary Figure S1. [file 41598_2021_82593_MOESM1_ESM.docx]
